# A series of homeopathic remedies-related severe drug-induced liver injury from South India

**DOI:** 10.1097/HC9.0000000000000064

**Published:** 2023-02-09

**Authors:** Arif Hussain Theruvath, Resmi Raveendran, Cyriac Abby Philips, Rizwan Ahamed, Jinsha K Abduljaleel, Ajit Tharakan, Sasidharan Rajesh, Philip Augustine

**Affiliations:** 1 Division of Complementary and Alternative Medicine (Homoeopathy), Department of Clinical Research, The Liver Institute, Center for Excellence in Gastrointestinal Sciences, Rajagiri Hospital, Aluva, Kerala, India; 2 Division of Complementary and Alternative Medicine (Ayurveda), Department of Clinical Research, The Liver Institute, Center for Excellence in Gastrointestinal Sciences, Rajagiri Hospital, Aluva, Kerala, India; 3 Clinical and Translational Hepatology & Monarch Liver Laboratory, The Liver Institute, Center for Excellence in Gastrointestinal Sciences, Rajagiri Hospital, Aluva, Kerala, India; 4 Gastroenterology and Advanced GI Endoscopy, Center of Excellence in GI Sciences, Rajagiri Hospital, Chunangamvely, Aluva, Kerala, India; 5 Interventional Hepatobiliary Radiology, The Liver Institute, Center of Excellence in GI Sciences, Rajagiri Hospital, Chunangamvely, Aluva, Kerala, India

## Abstract

Homeopathic remedies are highly diluted formulations without proven clinical benefits, traditionally believed not to cause adverse events. Nonetheless, published literature reveals severe local and non–liver-related systemic side effects. We present the first series on homeopathy-related severe drug-induced liver injury (DILI) from a single center.

**Methods:**

A retrospective review of records from January 2019 to February 2022 identified 9 patients with liver injury attributed to homeopathic formulations. Competing causes were comprehensively excluded. Chemical analysis was performed on retrieved formulations using triple quadrupole gas chromatography-mass spectrometry and inductively coupled plasma atomic emission spectroscopy.

**Results:**

Males predominated with a median age of 54 years. The most typical clinical presentation was acute hepatitis, followed by acute on chronic liver failure. All patients developed jaundice, and ascites were notable in one-third of the patients. Five patients had underlying chronic liver disease. COVID-19 prevention was the most common indication for homeopathic use. Probable DILI was seen in 77.8%, and hepatocellular injury predominated (66.7%). Four (44.4%) patients died (3 with chronic liver disease) at a median follow-up of 194 days. Liver histopathology showed necrosis, portal and lobular neutrophilic inflammation, and eosinophilic infiltration with cholestasis. A total of 29 remedies were consumed between 9 patients, and 15 formulations were analyzed. Toxicology revealed industrial solvents, corticosteroids, antibiotics, sedatives, synthetic opioids, heavy metals, and toxic phyto-compounds, even in ‘supposed’ ultra-dilute formulations.

**Conclusion:**

Homeopathic remedies potentially result in severe liver injury, leading to death in those with underlying liver disease. The use of mother tinctures, insufficient dilution, poor manufacturing practices, adulteration and contamination, and the presence of direct hepatotoxic herbals were the reasons for toxicity. Physicians, the public, and patients must realize that Homeopathic drugs are not ‘gentle placebos.’

## Graphical abstract

**Figure:**
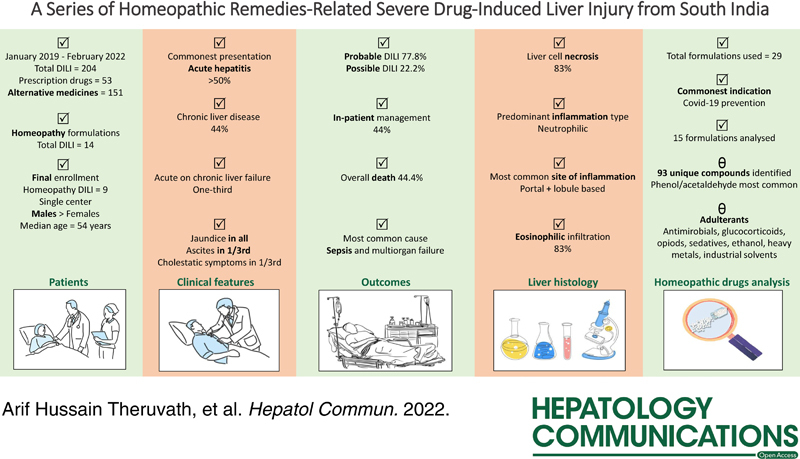

## INTRODUCTION

Homeopathy, an alternative system of medicine developed in Germany more than 2 centuries ago, is based on 3 unfounded theories, such as “like cures like”, “the lower the dose of medication, the greater its effectiveness (potentization)”, and “treatments to balance the dynamic derangements of the spirit-like power (the vital principle) that animates all humans.”^1^ In India, homeopathy is recognized as a distinct medical system by means of the Homeopathy Central Council Act 1973. Homeopathic remedies are promoted for viral diseases, allergic conditions, skin disorders, behavioral problems, and several chronic illnesses. The Indian Government’s official website, The National Health Portal, specifies that “the strength of homeopathy lies in its effectiveness on certain clinical conditions for which there is less treatment in other medical systems and that it can be utilized as a standalone treatment for hormonal disorders, pain, and palliative care and infertility.” (https://www.nhp.gov.in/homeopathy_mty). The National Health Service, UK, defines Homeopathy as a “treatment” based on highly diluted substances, which practitioners claim can cause the body to heal itself. Nonetheless, after extensive investigation into its effectiveness, scientific studies conclude that no good-quality evidence exists that homeopathy is effective as a treatment for any health condition. The National Health Service recommended that general practitioners and other prescribers stop providing homeopathy to patients (https://www.nhs.uk/conditions/homeopathy/). The National Center for Complementary and Integrative Health at the National Institutes of Health, USA, specifies that there’s little evidence to support homeopathy as an effective treatment for any specific health condition (https://www.nccih.nih.gov/health/homeopathy). This is because, as per classical homeopathy, the most potent medicines are the highly diluted ones. Some of these medications are exceedingly diluted, and no active molecule of the mother compound (starting material for dilution) exists in the formulation to promote clinical benefits.^2^ Nonetheless, the National Center for Complementary and Integrative Health also mentions that some homeopathic formulations may contain substantial amounts of active ingredients that could cause side effects and drug interactions. A systematic review of the published case reports and case series on the adverse impact of homeopathy revealed various direct toxic events such as allergic and immune-mediated reactions, heavy metals (Arsenic, Thallium), inorganic (bromate), organic (aconite, kerosene, xylitol), and phytochemical (saponin glycosides) intoxication, hypersensitivity reactions, drug interactions, alcohol, hormone, and recreational agent contaminations. All of these resulted in clinical events such as anaphylaxis, severe skin-related adverse events such as erythroderma and drug reaction with eosinophilia and systemic symptoms syndrome, systemic events such as severe gastrointestinal symptoms, acute pancreatitis, cardiac arrest, acute nephritis, bladder malignancy, dyselectrolytemia, delirium, agitation, and encephalopathy leading to coma.^3^ There is no considerable published evidence of adverse events associated with homeopathy in the context of drug-induced liver injury (DILI) in the current literature. In this study, we report on the clinical features, histologic patterns, and chemical and toxicology analysis associated with homeopathic-remedies-related DILI in a considerable cohort of patients from a tertiary care liver institute.

## METHODS

We accessed electronic hospital records from January 2019 to February 2022 to identify all patients diagnosed with DILI. Acute hepatitis was considered when liver injury due to a drug, remedy, or supplement resulted in the elevation of aspartate aminotransferase (AST) or alanine aminotransferase (ALT) more than 5 times the upper limit of normal with or without jaundice (total bilirubin >3.0 mg/dL).^4^ Cirrhosis was diagnosed on history and clinical examination following radiologic or histologic evidence. Acute decompensation due to DILI was considered when there was an acute deterioration in the liver function in a patient with stable cirrhosis, characterized by the development of acute hepatitis and ascites or hepatic encephalopathy (HE) without prior history of these complications, excluding those with acute on chronic liver failure (ACLF).^5^ For the ease of inclusion, the Asia-Pacific Association for Study of Liver definition of ACLF, that is, “acute hepatic insult manifesting as jaundice [serum bilirubin ≥ 5 mg/dL and coagulopathy (international normalized ratio or INR) ≥ 1.5, complicated within 4 weeks by clinical ascites and/or HE] was utilized.^6^ Hepatic encephalopathy (HE) was graded as per the West Haven criteria. Ascites were graded as no ascites, mild (grade 1, detected on ultrasonography), moderate (grade 2, shifting dullness), and severe (grade 3, fluid thrill) based on the clinical and radiologic evaluations.^7^,^8^ In all cases, a thorough diagnostic workup was performed. Other well-recognized competing causes for liver injury were excluded based on laboratory tests, viral serologies, autoimmune markers, diagnostic imaging, and liver histology where possible. Patients with clinically identifiable triggers for an acute event and complementary and alternative medicines (CAMs) other than homeopathic formulations were excluded from the final analysis. DILI, due to conventional prescription medications and other CAMs utilized in the Indian sub-continent, such as Ayurveda, Siddha, and Unani herbal supplements, folk medicines, and Tibetan drugs, as well as herbal and dietary supplements, were thus excluded. We also excluded all the patients who were concomitantly taking these medicines or any known or potentially hepatotoxic agents along with homeopathic remedies within 3 months preceding the diagnosis of DILI. Those with significant alcohol consumption, acute and chronic viral hepatitis, and classic autoimmune hepatitis were also excluded. The type of liver injury was determined using the R ratio. R ratio less than 2 was defined as cholestatic, between 2 and 5 as mixed, and greater than 5 as hepatocellular.^9^ The causality assessment in all cases was based on the updated Roussel-Uclaf Causality Assessment Method score, which assigns scores that range from +15 to −3, with highly probable DILI requiring a score of >8, probable requiring a score of 6–8, possible requiring a score of 4–6, and unlikely requiring a score of 1–3.^10^ A drug rechallenge to diagnose ‘definite’ DILI was not undertaken, except when the patient warranted it or was inadvertently exposed, given ethical concerns, patient protection, and the noncritical category of implicated medications.^11^ We followed and adhered to the minimal elements for reporting DILI as described by Agarwal et al^12^ to ensure completeness of the diagnostic workup and the categorical diagnosis of DILI. In those patients who consented, percutaneous or transjugular liver biopsies, depending on the presence or absence of ascites or significant coagulation failure, were undertaken to study histopathological patterns of Homeopathic DILI. All patients were followed up for the resolution of DILI and survival, death, or liver transplantation. Where possible, the implicated homeopathic remedies were retrieved for further chemical and toxicology analysis. Heavy metal concentration was determined by an inductively coupled plasma atomic emission spectrometer (ICP-AES; Thermo Electron, IRIS Intrepid II XSP Duo). The methodology, chemical standards, reagents, and vials were acquired per standards set by the United States Environmental Protection Agency (USEPA), method 5021A, 8015, 8021, and 8260. Complete analysis qualitative analyses were performed using triple-quadruple gas chromatography coupled to tandem mass spectrometry method (GC/MS-MS; Varian Saturn 2200; Agilent Technologies). Headspace analysis and purge and trap methods were utilized for solid and liquid samples, respectively.^13^ Statistical analysis was performed using MedCalc Statistical Software. Data were given as mean and SD or median and range as applicable. The study was performed to conform to the Helsinki Declaration of 1975, as revised in 2000 and 2008, concerning human and animal rights and was approved by the hospital ethical review board.

## RESULTS

### Patient Screening and Inclusion

**FIGURE 1:**
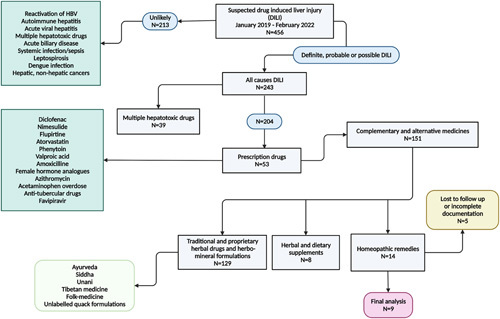
Patient inclusion consort flow diagram.

Initial screening of the patient records from January 2019 to February 2022 revealed 456 patients with suspected DILI. After excluding 213 patients with confirmed competing causes for acute hepatitis, 243 patients with definite, probable, or possible DILI underwent screening for further inclusion. Thirty-nine patients were excluded due to suspected DILI due to multiple hepatotoxic agents—both conventional prescription drugs and CAMs. Of the remaining 204 patients, 53 were found to have DILI due to prescription drugs, including non–steroidal anti-inflammatory drugs, lipid-lowering agents, anti-epileptics, antimicrobials, hormonal analogues, and antipyretics. Finally, 151 cases of DILI secondary to CAMs were identified. Of these, 129 cases were due to traditional and proprietary herbal drugs and herbo-mineral formulations and 8 cases due to herbal and dietary supplements were excluded, yielding 14 patients with homeopathic-remedies-related to DILI. After excluding 5 patients who were lost to follow-up or with incomplete documentation, ultimately, 9 patients with homeopathic-remedies-related to DILI were included for final analysis (Fig. 1).

### Patient Demographics and Baseline Clinical and Investigational Characteristics

Males predominated (n=6, 66.7%) with a median age (n=9) of 54 years. The commonest clinical presentation was acute hepatitis (n=5, 55.6%), followed by ACLF in one-third and acute decompensation of cirrhosis in 1 patient. All patients had jaundice at the presentation. Ascites and cholestatic symptoms were notable in one-third, while HE was absent at index presentation. Features of metabolic syndrome in the form of diabetes mellitus, systemic hypertension, and obesity were present in 2 (22.2%), 1 (11.1%), and 4 (44.4%) patients, respectively. None of the 9 patients had hypothyroidism or cardiovascular disease. In addition, 3 patients each had other underlying diseases such as Gilbert’s syndrome, allergic bronchitis, and hepatocellular carcinoma, respectively. Four patients (44.4%) were known to have underlying chronic liver disease (CLD), and 1 was diagnosed to harbor CLD during the evaluation of DILI. The etiology of CLD was NAFLD in 4 (n=5, 80%) patients. Admission for in-patient management at first contact was required in 4 (44.4%) patients with Homeopathic remedy-induced liver injury. One patient required intensive unit care management at first contact. Overall, admission at any time during the treatment and follow-up was required in 5 (55.6%) patients. In the total cohort, 4 (44.4%) patients underwent a one-time in-hospital stay. At the same time, 1 each encountered 2, 4, and 5 hospital admissions during the treatment for homeopathic remedy-induced liver injury. Complete outpatient-based management was required in only 2 (22.2%) patients in the cohort. The median follow-up from the first consultation or admission was 194 (25–75 P, 66–297.5) days. The mean (n=9) total bilirubin, median AST and ALT, mean prothrombin time, and model for end-stage liver disease score (in cirrhotics, n=4) was 10.1±6.4 mg/dL, 466 U/L, 219 U/L, 21.9±10.8 seconds, and 30.3±1.5 respectively. The complete baseline investigational details are shown in Table 1. The updated Roussel-Uclaf Causality Assessment Method score demonstrated ‘probable’ in 7 (77.8%) and ‘possible’ DILI in 2 (22.2%) patients, while the R ratio describing the type of liver injury was predominantly hepatocellular (n=6, 66.7%). The mean immunoglobulin G level was 15.5±2.2 g/L, and none of the patients were positive for autoimmune hepatitis-related autoantibodies. During a median follow-up of 194 days, 1 patient (n=5/9) each developed complications associated with progressive liver failure such as HE, acute kidney injury, recurrent ascites, sepsis, or a combination of these. Four (44.4%) patients died on follow-up. The commonest cause of death was sepsis leading to multiple organ failure in 75%, and progressive liver failure leading to multiple organ failure in the rest. The patient-wise salient investigations, clinical features, and pertinent discussions on competing risks and outcomes are shown in Supplementary tables 1 and 2, http://links.lww.com/HC9/A157, http://links.lww.com/HC9/A158.

**TABLE 1:** Investigational characteristics of the patients with Homeopathic drug-induced liver injury in the whole cohort

	N	Minimum	Maximum	Mean	Median	SD	25–75 P
Age (y)	9	26	70	48.4	54	17.6	32.2–65.7
Hemoglobin (g/L)	9	9.5	16	12.2	11.6	2.4	10–14.2
Total counts (×1000 per cubic mm)	9	4.3	12.6	6.7	5.6	2.7	4.6–7.8
Platelet counts (×1000 per cubic mm)	9	60	246	151.6	130	72.7	87.5–220.3
Total bilirubin (mg/dL)	9	3.6	23.8	10.1	8.9	6.4	4.8–13.3
Direct bilirubin (mg/dL)	9	2	18.1	7.1	6.4	5.1	2.6–9.5
Aspartate aminotransferase (U/L)	9	80	2007	610.1	466	618.7	188.5–920
Alanine aminotransferase (U/L)	9	54	2655	582.4	219	828.7	82–703.3
Alkaline phosphatase (U/L)	9	88	226	150.6	134	44.4	124.7–176.7
Gamma-glutamyl transferase (U/L)	9	92	301	185.7	195	74.3	101–243
Total protein (g/L)	9	6.7	8	7.2	7.2	0.4	6.8–7.5
Albumin (g/L)	9	2.5	4.2	3.4	3.6	0.7	2.7–4
Globulin (g/L)	9	2.8	5.2	3.9	4.1	0.8	3.2–4.4
Blood urea (mg/dL)	9	12	34	19.6	18	6.4	16–21.3
Creatinine (mg/dL)	9	0.6	1.5	0.9	0.8	0.3	0.7–1.13
Sodium (meq/L)	9	124	145	135.2	137	7.9	128.5–142.5
Potassium (meq/L)	9	3.8	5.2	4.3	4.2	0.5	3.9–4.5
Total immunoglobulin G (g/L)	9	12	18.4	15.5	15.9	2.2	13.7–17.13
Model for end-stage liver disease score	4	29	32	30.3	30	1.5	29–31.5
Total follow-up from the first consultation (days)	9	27	382	192	194	133.4	66–297.5
Total number of days of Homeopathy consumed	9	30	90	56.4	62	23	32–72.5
Time to development of abnormalities (in days)	9	15	92	55.6	65	26.8	29.5–74
Roussel-Uclaf Causality Assessment Method Score	9	5	8	6.8	7	0.9	6.0–7.3
Prothrombin time (seconds)	9	13.2	41.2	21.9	15.1	10.8	13.3–30.2
International normalized ratio	9	0.98	2.9	1.6	1.2	0.7	0.9–2.2
Number of Homeopathy remedies taken	9	1	6	3.2	3	1.6	2–4.2

### Liver Histopathology

**FIGURE 2:**
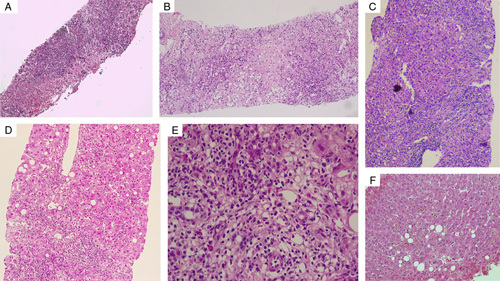
Representative liver histopathology photomicrographs of patients who developed Homeopathic remedies related liver injury. (A)Bridging necrosis with moderate to severe portal and lobular inflammation (haematoxylin and eosin stain, ×10),(B) Confluent necrosis with mixed type inflammation consisting of neutrophilic, lymphocytic and eosinophilic infiltration (haematoxylin and eosin stain, ×20),(C) severe lymphoplasmacytic and neutrophilic inflammation in a patient with cirrhosis with areas of spotty necrosis (haematoxylin and eosin stain, ×40), (D)predominantly portal-based neutrophilic and lymphocytic inflammation with interface hepatitis with steatosis (haematoxylin and eosin stain, ×40), (E) commonest inflammation notable in patients was moderate to severe mixed cell (neutrophilic, lymphocytic, few plasma cells, and eosinophilic) type, in addition ballooning is notable (haematoxylin and eosin stain, ×200), (F) moderate to severe cholestasis (intracanalicular and hepatocytic) [haematoxylin and eosin stain, ×100].

A liver biopsy was performed on 6 (66.7%) consenting patients. Biopsy was not contemplated in a patient with ACLF due to homeopathic DILI and underlying hepatocellular carcinoma. Of those who underwent biopsy (n=6), 4 (66.7%) were through the transjugular route, while the rest were performed percutaneously. Necrosis was notable in 5 (83.3%) patients. Three patients (60%) had confluent/bridging necrosis, while 1 each had focal/spotty and submassive necrosis, respectively. The predominant inflammation pattern was neutrophilic or neutrophilic and lymphocytic (mixed) in 50% each, and the dominant inflammation type was both lobular and portal-based in 5 (83.3%) patients. Ballooning of hepatocytes was notable in 5 (83.3%) and cholestasis in 4 (66.7%) patients; the latter predominantly (n=2, 50%) of the mixed (both hepatocellular and canalicular) type. Eosinophils were remarkable in 5 (83.3%) patients, and 4 (80%) had moderate severity of eosinophilic infiltration. Similarly, interface hepatitis of predominantly moderate severity was also significant in 83.3% and steatosis in 66.7% of the biopsy samples analyzed. Fibrosis was pronounced in 5 patients (83.3%) in whom grades 1, 2, and 4 (cirrhosis) were remarkable in 2 (33.3%), 1 (16.7%), and 2 (33.3%) patients, respectively. The representative liver biopsies are shown in Figure 2.

### Homeopathic Remedies

Among 9 patients, the total number of homeopathic remedies consumed totalled 29, ranging from single remedy use to a maximum of 6 remedies (median 3). Patients consumed labelled, unlabelled, or both (each n=3, 33.3%) types of homeopathic formulations. All homeopathic formulations were prescribed or dispensed by trained and registered homeopaths. The consumed remedies were solid formulations in 2 (22.2%), liquid in 1 (11.1%), and both in 6 (66.7%) patients. The most common reason for homeopathy use was to prevent novel coronavirus disease (COVID-19) in 4 (44.4%) patients. Other reasons included atopic dermatitis and allergic cough, dyspepsia, arthritis, headache, Gilbert’s syndrome, hepatocellular carcinoma, penile lichen sclerosus, excessive hair fall, gall bladder, and renal stones. Homeopathic formulations were consumed for a median of 62 (minimum 30, maximum 90) days. The development of liver-related symptoms/abnormalities was at a median of 65 (minimum 15, maximum 92) days. Formulations were either classic Homeopathy or proprietary non-classic products made by private manufacturers. These also included raw mother tinctures, labelled and unlabeled, and classic formulations prepared by registered homeopathic practitioners without labels or content disclosures in the form of globules or powders packed in paper covers.

**FIGURE 3:**
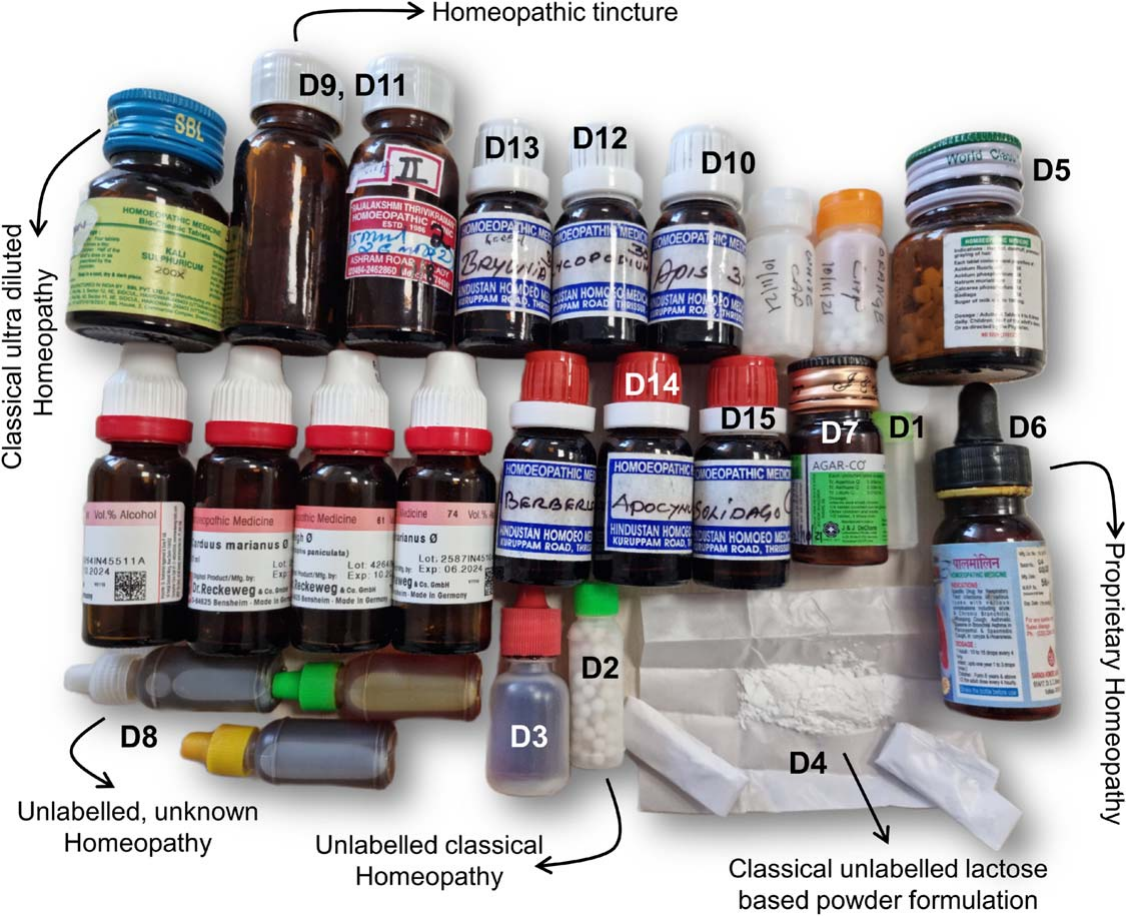
Various types of retrieved Homeopathic remedies. The letter-numbered remedies were sent for chemical and toxicology analysis. D1, D2, D3 and D6 (Covid prevention), D4 (gall bladder stones), D5 (excessive hair fall), D7 (headache/migraine), D8 (Gilbert’s syndrome), D9 and D11 (kidney stones), D10 (atopic dermatitis), D12 (dyspepsia), D13 (flu-like illness), D14 (allergic cough), D15 (arthritis pains).

**FIGURE 4:**
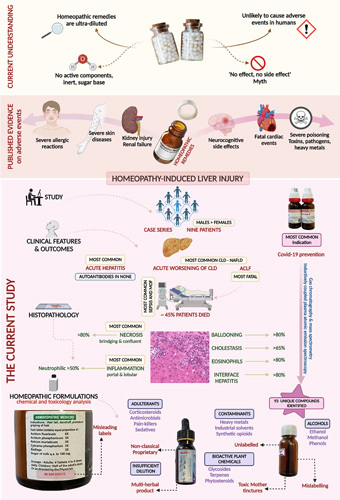
Infographic summary of the study.

We retrieved 25 homeopathic remedies (Fig. 3), of which 15 were found in sufficient quantity for chemical and toxicology analysis. We identified 93 unique compounds in 15 homeopathic remedies, including supposed ultra-diluted formulations on analysis. A total of 156 compounds, including repeats, were identified from 15 samples. The total number of compounds present in a formulation ranged from a minimum of 1 to a maximum of 23. The most commonly identified compounds were 2,4 di-tert-butyl-phenol nd acetaldehyde, which featured in 7 (46.7%) samples each. Antibiotics, including gentamycin and paromomycin, were identified in 6 (40%) formulations. Homeopathic formulations were also found to contain glucocorticoids (betamethasone), benzodiazepines (nordiazepam), phytosteroids (diosgenin, sarasapogenin), and a variety of industrial solvents such as benzene, butane, aldehyde, and carboxylic acid derivatives and sulfhydryl compounds. Potent synthetic opioids (normethadone), glucosides, glycosides, phenols, terpenes, heavy metal compounds [detectable arsenic (0.18 ppm), cobalt, chromium, zirconium, manganese, lithium, titanium dioxide, and vanadium oxysalts] were also identified on chemical analysis. Three liquid formulations contained high ethanol levels (15.8%, 18.3%, and 19.5% v/v), and 1 also had traces of methanol 0.001% v/v) (Fig. 4). The results of the exhaustive analysis are shown in Supplementary document 3, http://links.lww.com/HC9/A159.

## DISCUSSION

The current study is the first to describe a series of patients with Homeopathic formulations-induced liver injury. We described patterns of homeopathic remedies used within a large cohort of patients with DILI and further identified and characterized a group of patients who developed DILI related to homeopathy (Hom-DILI) use based on validated causality tools after carefully excluding competing causes. The pertinent features in our patients with Hom-DILI included jaundice at index presentation in all and ascites notable in one-third; more than half of patients had underlying CLD and probable Hom-DILI was notable in ~80% of patients, with rechallenge positive in 1. Mortality was seen in 44% of those who developed Hom-DILI, the majority with underlying CLD. Classic homeopathy is considered safe due to the extreme ultra-dilution process performed during the preparation of the remedies, which makes the final formulation devoid of any active compound. Nonetheless, this is far from reality. A recent meta-analysis of multiple observational studies on homeopathy revealed that adverse events associated with homeopathic remedies were significantly higher when compared with controls. The majority of adverse events included gastrointestinal disorders, headache/dizziness, allergic reactions, dermatitis, and upper respiratory tract infections.^14^

A detailed systematic review of homeopathic remedies-induced adverse events from published case reports and case series by Posadzski and colleagues showed that severe side effects, some leading to fatality, are possible with classic and unspecified homeopathic formulations. The total number of patients included was 1159, of which 1142 suffered adverse events directly related to homeopathy. The direct adverse events had acute pancreatitis, severe allergic reactions, arsenical keratosis, bullous pemphigoid, neurocognitive disorders, sudden cardiac arrest and coma, severe dyselectrolytemia, interstitial nephritis, kidney injury, thallium poisoning, syncopal attacks, and focal neurological deficits as well as movement disorders. Fatal events involved advanced renal failure requiring dialysis, toxic polyneuropathy, and quadriparesis. The duration of adverse events ranged from a few hours to 7 months, and 4 patients died. The authors state that in most cases, the mechanism of action for side effects of homeopathy involved allergic reactions or the presence of toxic substances—the use of strong mother tinctures, drug contaminants, adulterants, or poor manufacturing (incorrect dilutions).^3^

The present study is the first to report a series of patients with hepatobiliary adverse events associated with homeopathic formulations. In our cohort of patients, the clinical presentation was either in the form of acute hepatitis, acute worsening of CLD, or ACLF. None of the patients presented with features of acute liver failure or progressed to chronic liver injury. This contrasts with herb-induced liver injuries due to Ayurvedic herbal supplements or traditional Chinese medicine in which the progression to acute liver failure or chronic herb-induced liver injuries is well-described.^13^,^15^,^16^ In classic Homeopathic practice, most remedies are too dilute to have clinical effects and hence the absence of adverse effects. Nonetheless, many Homeopathic practitioners prescribe mother tinctures, which are pure, alcohol-based forms of heavy metals or herbals that are poisonous and toxic, unconventional products (such as proprietary multi-ingredient formulations), and insufficiently diluted remedies. Our study also showed similar findings when retrieved formulations were analyzed. We identified 156 unique compounds, including heavy metals, industrial solvents, alcohols, steroids, antimicrobials, sedatives, and bioactive plant compounds, such as diterpenes, isoquinolines, furanoids, terpenes, glycosides, and sterols, even in supposedly “ultra-diluted” formulations. Many bioactive plant compounds, such as terpenes and glycosides and organic and inorganic solvents, have been linked to severe liver injury.^17^–^20^ This demonstrates that adverse liver-related events due to homeopathy mainly were related to the use of concentrated forms of remedies, adulteration, contamination, insufficient dilution, and the presence of alcohol. Traditionally, homeopathic principles, teaching, and practice are void of pharmacovigilance because the concept of adverse drug reaction is lacking. Instead, homeopaths describe an adverse event as ‘homeopathic aggravations’ —where there is a worsening of the underlying disease or new onset of symptoms that are considered part of the ‘healing process.’ In this context, a large body of published evidence in homeopathy lacks descriptions and discussions on general and specific adverse events directly related to the treatments.^21^

Contamination and adulteration, or the use of toxic forms of homeopathic remedies leading to adverse events in humans, including fatality, is well-described in the literature. Our paper also provides striking evidence that such formulations can cause avoidable liver disease burden in the general and patient population.^22^,^23^ An intriguing aspect of our study was the absence of autoimmune hepatitis-related antibodies in all the patients, even those with elevated serum immunoglobulin levels. This contradicts liver injury due to Ayurvedic formulations in which specific herbs such as Giloy (Tinospora cordifolia) have been identified to produce de-novo autoimmune hepatitis or trigger silent autoimmune hepatitis in predisposed patients.^24^ The present study also provides insights into liver histopathology associated with Hom-DILI, an aspect missing in the current literature on complementary and alternative medicine-related DILI. In our patients, hepatocyte necrosis of the bridging or confluent type was the most typical finding and inflammation predominantly of the neutrophilic type. The inflammation was commonly portal as well as lobule-based with remarkable eosinophilic infiltration. None of the patients had the features of autoimmune hepatitis on biopsy, and cholestasis was featured often. The variable, undefining findings on liver biopsy in patients with Hom-DILI are similar to current published literature on DILI and herb-induced liver injury. This is probably due to the heterogeneous nature of ingredients that form part of unstandardized homeopathic formulations. Akin to published literature, Hom-DILI can also present (clinical as well as histopathological) in various forms, mimicking other common forms of liver disease.^25^–^27^

Our study has various strengths and weaknesses. Ours is the first series to describe homeopathic formulations-induced liver injury in a cohort of patients with and without underlying liver disease and analyze chemical and toxicological properties of homeopathic remedies retrieved from affected patients. We also provide pilot descriptions of liver histopathology in Hom-DILI. Our paper negates the myth that homeopathic formulations are ultra-diluted without adverse effects. The concerns that plague drug regulation in the mainstream and other alternative forms of medicine also afflict the homeopathy industry. Using concentrated hepatotoxic formulations, unconventional combinations, multiple remedies in a single patient, and poor manufacturing practices leading to contamination and adulteration with known liver toxic agents form the basis of Hom-DILI. The major limitation of our study was that it was retrospective in nature, from a single center, and featured a small group of patients. We identified cases for potential inclusion based on ALT >5 times the upper limit of normal. The United States DILIN criteria for assessing drug-induced liver injury utilizes broader criteria, including serum alkaline phosphatase >2 times the upper limit of normal, or jaundice or coagulopathy with elevated ALT or AST. As most patients we included were referred from elsewhere for expert evaluation, we believe there may have been cases of Hom-DILI, particularly cholestatic type, that were missed due to referral bias and the study’s predefined inclusion criteria and retrospective nature.

Liver biopsy could not be performed in all patients, all homeopathic formulations could not be identified and retrieved, and all those retrieved could not be analyzed. The exact formulation or component of a formulation that caused Hom-DILI could not be placed in the majority as multiple remedies were consumed by 1 patient, some formulations contained numerous herbal ingredients, some were unlabelled, and also due to the changes in prescriptions on various follow-ups. Nonetheless, excluding competing causes strongly supported Homeopathic remedies as the most probable cause of DILI in our group of patients and the liver biopsy strongly supported DILI due to homeopathic agents. Furthermore, the exhaustive chemical and the toxicologic analysis of retrieved agents provided pertinent evidence that homeopathic remedies were, in all likelihood, the cause of liver injury in our group of patients.

## CONCLUSION

It is believed that classic homeopathic remedies do not contain active substances and work on a placebo effect. They hence do not have clinical effects as well as direct adverse events in the form of organ damage as they mostly contain inert sugar in water or alcohol vehicles. However, contrary to current beliefs, we demonstrate that homeopathic formulations, both traditional and proprietary, can potentially cause liver toxicity due to direct or indirect toxins. These toxins feature in the formulations due to the direct use of pure tinctures, inadequate dilution process, and contamination and adulteration due to poor manufacturing practices. Physicians caring for patients with acute onset liver disease or acute worsening of chronic liver disease must be aware that homeopathic remedies, once considered an ineffective placebo, could play a central role in promoting severe liver injury and a modifiable liver disease burden within the community.

## Supplementary Material
