# Supplementary material for: A series of homeopathic remedies-related severe drug-induced liver injury from South India
Source: Hepatol Commun. 2023 Feb 9;7(3):e0064. doi: 10.1097/HC9.0000000000000064 (PMC9916127; doi:10.1097/HC9.0000000000000064)
Supplement: Supplementary file 1 [file hc9-7-e0064-s001.docx]

| **Supplementary table 1:** Investigational features of individual patients | | | | | | | | | | | | | | | | | | | | | | | |
| --- | --- | --- | --- | --- | --- | --- | --- | --- | --- | --- | --- | --- | --- | --- | --- | --- | --- | --- | --- | --- | --- | --- | --- |
| **Patient** | **Age/Sex** | **Clinical** | **Hb** | **TC** | **PC** | **TB** | **DB** | **AST** | **ALT** | **ALP** | **GGT** | **TP** | **Alb** | **Glob** | **PT** | **INR** | **Urea** | **Creat** | **Na** | **K** | **Admission MELD** | **IgG (Total)** |  |
| **1** | 65/F | ACLF | 10.7 | 5.5 | 130 | 10 | 7.1 | 478 | 82 | 152 | 242 | 6.9 | 2.8 | 4.1 | 23 | 1.75 | 18 | 0.6 | 124 | 4.3 | 29 | 17.8 |  |
| **2** | 26/F | AH | 13.1 | 6.4 | 246 | 3.8 | 2.1 | 466 | 884 | 226 | 301 | 7.2 | 4.1 | 3.1 | 13.8 | 0.99 | 12 | 0.8 | 142 | 5.2 | - | 12 |  |
| **3** | 54/F | AH | 11.6 | 7.1 | 220 | 8.9 | 6.4 | 1022 | 643 | 134 | 98 | 6.8 | 4 | 2.8 | 13.4 | 1 | 22 | 1.1 | 145 | 3.9 | - | 13.4 |  |
| **4** | 54/M | ACLF | 9.8 | 4.3 | 89 | 5.2 | 2.8 | 202 | 54 | 127 | 102 | 6.7 | 2.5 | 4.2 | 35.2 | 2.74 | 18 | 0.7 | 127 | 4.2 | 29 | 16.9 |  |
| **5** | 27/M | AH | 14.8 | 4.6 | 221 | 3.6 | 2 | 80 | 219 | 88 | 92 | 7.4 | 4.2 | 3.2 | 13.2 | 1 | 13 | 1 | 144 | 4.3 | - | 15.9 |  |
| **6** | 68/M | AH | 14 | 5.6 | 214 | 7.8 | 5.9 | 886 | 462 | 165 | 208 | 7.3 | 3.9 | 4.4 | 13.3 | 0.98 | 21 | 1.2 | 139 | 3.8 | - | 18.4 |  |
| **7** | 70/M | ACLF | 9.5 | 12.6 | 83 | 15 | 9.9 | 148 | 161 | 212 | 195 | 6.8 | 2.9 | 3.9 | 41.2 | 2.92 | 17 | 0.8 | 129 | 4 | 32 | 14.6 |  |
| **8** | 34/M | AH | 16 | 4.6 | 60 | 23.8 | 18.1 | 2007 | 2655 | 133 | 246 | 8 | 3.6 | 4.4 | 15.1 | 1.2 | 21 | 0.6 | 137 | 5.1 | - | 13.8 |  |
| **9** | 38/M | AD | 10.1 | 9.8 | 101 | 12.8 | 9.4 | 202 | 82 | 118 | 187 | 7.8 | 2.6 | 5.2 | 28.6 | 2.1 | 34 | 1.5 | 130 | 3.9 | 31 | 16.8 |  |
| Footnote: F – female, M – male, ACLF – acute-on-chronic liver failure, AH – acute hepatitis, AD – acute decompensation, Hb – hemoglobin (g/L), TC – total leucocyte count (x 10^3^ per L), PC – platelet count (x 10^3^ per L), TB – total bilirubin (mg/dl), DB – direct bilirubin (mg/dl), AST (peak) – aspartate aminotransferase (U/L), ALT (peak) – alanine aminotransferase (U/L), ALP – alkaline phosphatase (U/L), GGT – gamma-glutamyl transpeptidase (U/L), TP – total protein (g/L), Alb – serum albumin (g/L), Glob – serum globulin (g/L), PT – prothrombin time (in seconds), INR – international normalized ratio, creat – serum creatinine (mg/dl), Na – serum sodium (mmol/L), K – serum potassium (mmol/L), MELD – model for end stage liver disease score, IgG – total immunoglobulin G level. | | | | | | | | | | | | | | | | | | | | | | | |
|  | | | | | | | | | | | | | | | | | | | | | | | |
