# Supplementary material for: A series of homeopathic remedies-related severe drug-induced liver injury from South India
Source: Hepatol Commun. 2023 Feb 9;7(3):e0064. doi: 10.1097/HC9.0000000000000064 (PMC9916127; doi:10.1097/HC9.0000000000000064)
Supplement: Supplementary file 2 [file hc9-7-e0064-s002.docx]

**Supplementary table 2:** Salient clinical features, outcomes and details of Homeopathic remedies consumed by nine patients in the case series

| **No** | **Patient** | **CLD etiology**  **Comorbidities** | **Reason for Homeopathy** | **Details of Homeopathy** | **Clinical course, treatment, and outcome** |
| --- | --- | --- | --- | --- | --- |
| **1** | 65/F | NAFLD  Diabetes | Liver cancer  BCLC-C | ***Type of Homeopathy***  Five types of marked but unlabelled products, including pills and tinctures, classical and proprietary formulations  ***Homeopathic formulation(s) implicated***  D5, D7, D12  ***Duration of therapy***  62 days  ***Onset of symptoms***  60 days  ***Onset of laboratory abnormalities***  66 days  ***Comments***  All remedies could not be retrieved  The medication implicated could not be identified as multiple Homeopathic remedies were utilized | ***Type of liver injury***  Cholestatic, unrelated to underlying cirrhosis or liver cancer (R = 1.13)  ***RUCAM score***  5 (possible)  ***Liver biopsy***  Not performed  ***Competing causes***  Progression of underlying liver cancer  ***Treatment***  Best supportive care  Albumin infusion, empirical antibiotics, intravenous N-acetyl cysteine, intravenous glutathione  ***Clinical course***  ACLF, progressive liver failure, multiple organ failure, death at 27 days from DILI diagnosis |
| **2** | 26/F | No CLD  Overweight | Atopic dermatitis | ***Type of Homeopathy***  Six types of marked but unlabelled products in the form of pills, liquids and powders  ***Homeopathic formulation(s)***  D10, D14, D15  ***Duration of therapy***  90 days  ***Onset of symptoms***  86 days  ***Onset of lab abnormalities***  86 days  ***Comments***  All remedies could not be retrieved  The medication implicated could not be identified as multiple Homeopathic remedies were utilized | ***Type of liver injury***  Hepatocellular (R = 14.3)  ***RUCAM score***  8 (probable)  ***Rechallenge***  Yes  Recurrence of symptoms and hepatitis  ***Liver biopsy***  Acute hepatitis, focal and spotty necrosis, mixed inflammation of portal and lobular regions, moderate eosinophilic infiltration, no fibrosis  ***Competing causes***  None  ***Treatment***  Weight-based corticosteroids for one month  Ursodeoxycholic acid 10mg/kg for one month  ***Clinical course***  Complete resolution of hepatitis  No recurrence beyond six months follow-up |
| **3** | 54/F | No CLD  No comorbidities | Covid-19 prevention | ***Type of Homeopathy***  Two types, one labelled Arsenicum Album 30C and the other unlabelled, both pills  ***Homeopathic formulation(s) implicated***  D2  ***Duration of therapy***  45 days  ***Onset of symptoms***  45 days  ***Onset of lab abnormalities***  48 days  ***Comments***  No other medications were consumed before or during the time Homeopathic remedies were used | ***Type of liver injury***  Hepatocellular (R = 27.8)  ***RUCAM score***  8 (probable)  ***Rechallenge***  Not performed  ***Liver biopsy***  Acute hepatitis with bridging necrosis with neutrophils, lymphocytes, and eosinophilic inflammation without fibrosis  ***Competing causes***  None  ***Treatment***  Weight-based corticosteroids tapered and stopped over six months  N-acetyl cysteine 1.2g/d for one month  ***Clinical course***  Complete resolution of hepatitis in three months on treatment, low dose immunosuppression maintained and stopped at six months  No recurrence beyond six months follow-up |
| **4** | 54/M | ALD | Covid-19 prevention | ***Type of Homeopathy***  Two types of unlabelled Homeopathic pills were provided in a paper cover marked Arsenicum Album 30C  ***Homeopathic formulation(s) implicated***  D1  ***Duration of therapy***  63 days  ***Onset of symptoms***  65 days  ***Onset of laboratory abnormalities***  65 days  ***Comments***  Retrieved remedies that were sent for analysis were unlabelled formulations  Other medications included beta blockers, low-dose diuretics, vitamin E and vitamin B supplements, and lactulose syrup that were ongoing for more than a year | ***Type of liver injury***  Cholestatic, unrelated to underlying cirrhosis or liver cancer (R = 1.7)  ***RUCAM score***  6 (probable)  ***Liver biopsy***  Not performed  ***Competing causes***  None  ***Treatment***  Best supportive care  Albumin infusion, empirical antibiotics, intravenous N-acetyl cysteine, ursodeoxycholic acid (10 mg/kg per day in divided dose)  ***Clinical course***  ACLF, resolution of cholestatic hepatitis within one month, but unstable decompensation with recurrent ascites for three months, recompensation at six months  The patient remained compensated at 243 days follow-up |
| **5** | 27/M | No CLD | Penile lichen sclerosus (also had incidental gall bladder stones) | ***Type of Homeopathy***  Four types of marked, but unlabelled products, including pills and powders, both classical and proprietary formulations  ***Homeopathic formulation(s) implicated***  D4, D13  ***Duration of therapy***  68 days  ***Onset of symptoms***  68 days  ***Onset of laboratory abnormalities***  70 days  ***Comments***  All remedies could not be retrieved | ***Type of liver injury***  Hepatocellular (R = 9.1)  ***RUCAM score***  7 (probable)  ***Liver biopsy***  Not performed  ***Competing causes***  None  ***Treatment***  N-acetyl cysteine and oral glutathione for one month  ***Clinical course***  Acute hepatitis resolved with drug withdrawal within one month  No recurrence of hepatitis was noted at 128 days of follow up |
| **6** | 68/M | NAFLD  Hypertension | Covid-19 prevention | ***Type of Homeopathy***  Arsenicum Album 30C  ***Homeopathic formulation(s) implicated***  D2  ***Duration of therapy***  32 days  ***Onset of symptoms***  28 days  ***Onset of laboratory abnormalities***  32 days  ***Comments***  The patient was not on other medications except vitamin E and telmisartan | ***Type of liver injury***  Hepatocellular (R = 15.8)  ***RUCAM score***  7 (probable)  ***Liver biopsy***  Lymphocytic, neutrophilic, and moderate plasmacytic inflammation affecting portal and lobular regions associated with moderate eosinophilic infiltration, moderate steatosis, grade 2 fibrosis, and mild interface hepatitis  ***Competing causes***  None  ***Treatment***  Immunosuppression – corticosteroid (first 6 months) and add-on azathioprine after 6 months, continued, low dose  ***Clinical course***  Immunosuppression was started after two weeks of drug withdrawal and did not improve hepatitis, even though jaundice resolved. Low-dose immunosuppression continued at a one-year follow-up when taper at six months led to a flare of hepatitis. |
| **7** | 70/M | NAFLD  Diabetes mellitus | Covid-19 prevention | ***Type of Homeopathy***  Two types of remedies, one marked Arsenicum Album 30C and the other proprietary  ***Homeopathic formulation(s) implicated***  D6  ***Duration of therapy***  86 days  ***Onset of symptoms***  86 days  ***Onset of laboratory abnormalities***  92 days  ***Comments***  The patient was on metformin, glimepiride, and acarbose for diabetes for five years  No other medications or supplements were used | ***Type of liver injury***  Mixed (R = 2.8)  ***RUCAM score***  7 (probable)  ***Liver biopsy***  Transjugular liver biopsy showed neutrophil and eosinophil-rich inflammation with extensive confluent and bridging necrosis.  ***Competing causes***  None  ***Treatment***  Best supportive care  Albumin infusion, empirical antibiotics, intravenous N-acetyl cysteine, ursodeoxycholic acid (10 mg/kg per day in divided dose)  ***Clinical course***  The patient presented with acute on chronic liver failure with unstable decompensation on follow-up with death at 276 days from diagnosis |
| **8** | 34/M | No CLD | Renal stones | ***Type of Homeopathy***  Two types of remedies, marked tinctures of Sarsaparilla Q and Berberis Q  ***Homeopathic formulation(s) implicated***  D9, D11  ***Duration of therapy***  32 days  ***Onset of symptoms***  30 days  ***Onset of laboratory abnormalities***  32 days  ***Comments***  The patient did not have underlying comorbidities and was not on other prescription drugs or supplements during this time. | ***Type of liver injury***  Hepatocellular (R=19.4)  ***RUCAM score***  7 (probable)  ***Liver biopsy***  Predominantly neutrophilic infiltration with extensive confluent necrosis.  ***Competing causes***  None  ***Treatment***  Corticosteroids (weight based)  Intravenous and oral N-acetyl cysteine, ursodeoxycholic acid (10 mg/kg per day in divided dose), and intravenous and oral glutathione  ***Clinical course***  Corticosteroids improved liver functions within 15 days to normal baseline levels.  On follow-up, the patient developed secondary aplastic anemia associated with the recurrence of acute hepatitis, which progressed to complete bone marrow failure  The patient died of sepsis 74 days after diagnosis. |
| **9** | 38/M | NAFLD  Obese | Gilbert’s syndrome | ***Type of Homeopathy***  Four types of Homeopathic remedies, all unlabelled  ***Homeopathic formulation(s) implicated***  D8  ***Duration of therapy***  30 days  ***Onset of symptoms***  15 days  ***Onset of laboratory abnormalities***  30 days  ***Comments***  The patient had underlying NAFLD-related liver cirrhosis  He was on vitamin E supplements and multivitamin B complex tablets four months before the start of Homeopathy | ***Type of liver injury***  Hepatocellular (R = 10.6)  ***RUCAM score***  6 (probable)  ***Liver biopsy***  Transjugular biopsy showed portal-based inflammation rich in neutrophils, eosinophils, and lymphocytes with cholestasis and interface hepatitis.  ***Competing causes***  None  ***Treatment***  Best supportive care  A short course of corticosteroids (stopped after two weeks), albumin infusion, empirical antibiotics, intravenous N-acetyl cysteine, ursodeoxycholic acid (10 mg/kg per day in divided dose)  ***Clinical course***  The patient developed acute decompensation of underlying cirrhosis  Clinical progression to acute on chronic liver failure  Died 42 days after diagnosis |

***For Homeopathic formulation(s) consumed, please see the corresponding codes in Figure 3. Marked formulation meant that the name of the formulation was handwritten by the prescriber on the drug without a standard label. Homeopathic remedies are traditionally not prescribed for diseases but for the ‘totality of symptoms.’ If a patient has multiple symptoms associated with a single disease, Homeopathic management considers symptoms to treat based on ‘like cures like’ and does not consider a final diagnosis.**
